# Influence of pregnancy related anthropometric changes on plantar pressure distribution during gait—A follow-up study

**DOI:** 10.1371/journal.pone.0264939

**Published:** 2022-03-11

**Authors:** Agata Masłoń, Agnieszka Suder, Marta Curyło, Barbara Frączek, Marcin Salamaga, Yuri Ivanenko, Wanda Forczek-Karkosz

**Affiliations:** 1 Section of Rehabilitation in Orthopaedics, Clinical Rehabilitation Institute, Faculty of Motor Rehabilitation, University of Physical Education, Krakow, Poland; 2 Section of Anatomy, Institute of Basic Sciences, Faculty of Motor Rehabilitation, University of Physical Education, Krakow, Poland; 3 Section of Rehabilitation in Internal Diseases, Clinical Rehabilitation Institute, Faculty of Motor Rehabilitation, University of Physical Education, Krakow, Poland; 4 Section of Sports Medicine and Human Nutrition, Institute of Biomedical Sciences, Faculty of Physical Education and Sport, University of Physical Education, Krakow, Poland; 5 Department of Statistics, Cracow University of Economics, Krakow, Poland; 6 Laboratory of Neuromotor Physiology, Santa Lucia Foundation, Rome, Italy; 7 Section of Biomechanics, Institute of Biomedical Sciences, Faculty of Physical Education and Sport, University of Physical Education, Krakow, Poland; West Virginia University, UNITED STATES

## Abstract

**Background:**

As foot constitutes the base of support for the whole body, the pregnancy-related anthropometric changes can result in adaptive plantar pressure alterations. The present study aimed to investigate how pregnancy affects foot loading pattern in gait, and if it is related to body adjustments to growing foetus that occur in the course of pregnancy.

**Methods:**

A prospective longitudinal study included 30 women. Three experimental sessions in accordance with the same procedure were carried out in the first, second and third trimesters of pregnancy. First, the anthropometric measures of the body mass and waist circumference were taken. Then walking trials at a self-selected speed along a ~6-m walkway were registered with the FreeMED force platform (Sensor Medica, Italy). Vertical foot pressure was recorded by the force plate located in the middle of the walkway.

**Findings:**

The correlation of individual foot loading parameters across different trimesters was relatively high. Nevertheless, our results revealed a longitudinal foot arch flattening with the strongest effect in late pregnancy (*P* = 0.01). The anthropometric characteristics also influenced the foot loading pattern depending on the phase of pregnancy. In particular, arch flattening correlated with the body mass in all trimesters (r≥0.44, *P*≤0.006) while the medial-lateral loading index correlated only in the first (r = 0.45, *P* = 0.005) and second (r = 0.36, *P* = 0.03) trimesters. Waist circumference changes significantly influenced dynamic arch flattening but only in the late pregnancy (r≥0.46, *P*≤0.004). In the third trimester, a small though significant increase in the right foot angle was observed (*P* = 0.01).

**Interpretation:**

The findings provided the characteristics of the relative foot areas loading throughout pregnancy. Growing abdominal size increases the risk of medial arch flattening, which can result in less stable gait. The observed increase in foot angle in late pregnancy may constitute a strategy to enhance gait stability.

## Introduction

During the course of pregnancy various physiological and hormonal changes take place, including constantly increasing body mass with its uneven distribution, relocation of the centre of gravity [1, 2], and increased joint laxity [3, 4]. The relative mass gain affects postural control of pregnant women [5–7] and causes gait kinematics adaptations [8]. The motor system has to adjust to pregnancy-related changes and adopt new control strategies to maintain postural and gait stability, including plantar pressure alterations [9].

Previous studies have reported some characteristics of foot loading during gait in pregnancy [10–16]. However, only few studies were of longitudinal character, including measurements in all 3 trimesters [9, 17–19]. The reported pregnancy-related changes in plantar pressure distribution during gait include: increased loading of forefoot in relation to rearfoot [9], or otherwise [18], greater loading of the lateral than medial part of the foot and increased loading of midfoot [19]. Also, some changes in the foot placement characteristics have been observed, such as a tendency to increase the step width to improve postural stability [17].

The most visible pregnancy-related body adjustment to developing foetus is mass gain and wider pelvis, which affects the kinematic features of gait [20]. Only in one study [14] the effect of mass gain on foot loading pattern during gait was analysed. The results of that study suggest that the growing body mass is compensated by a proportional increase of the muscle force to maintain an unchanged gait pattern. However, to our knowledge, there were no longitudinal studies on the relationship between anthropometric changes following foetus growth and plantar pressure distribution pattern during gait. Given that even small changes in the foot deformation and feedback from the foot cutaneous and muscular receptors affect both posture and gait [21, 22], longitudinal studies may further contribute to understanding important changes or adaptations in the gait pattern and stability that occur throughout pregnancy. Considering a reported high number of falls that occur in gestation period [6], it is of great importance to find out more about influencing factors. Therefore, the objective of this study was to characterise longitudinal changes in foot loading and their relationship to body adjustments to growing foetus that occur in the course of pregnancy. To achieve this goal we recorded and assessed alterations in plantar pressure distribution pattern in relation to mass gain and individual anthropometric changes in gravid women in the first, second and third trimesters. According to Sadeghi et al. it is crucial to accept that in the able-bodied population gait is asymmetrical, which can be associated with natural functional differences between the lower extremities [23]. That is why in this research we were looking for the gradual pregnancy-related changes of plantar pressure distribution pattern for each side separately to get insight into the process of adaptation of both feet. We hypothesised that in the course of pregnancy the progressive both feet longitudinal arch flattening will be observed. Moreover, we expected to observe the individual adaptations of feet (some alterations within the foot angle or/and relative foot loading), aiming to achieve more stable gait which would be dependable on the pregnancy period. Our longitudinal analysis gives a new insight into understanding the process of pregnancy-related gradual adaptations of foot loading pattern and their dependence on individually variable anthropometric factors.

## Materials and methods

### Subjects

The study was carried out in the Biomechanics Laboratory at the University of Physical Education in Krakow. In order to collect the study group, a three-years-long (2015–2018) recruitment process was conducted. The participants were informed about the project via personal contact but also using flyers distributed in hospitals or gynaecological clinics. For the women who volunteered to participate in the study, specific criteria were introduced before including them to the study group. The inclusion criteria comprised: age between 20 and 40 years, initial body mass index (BMI) range between 18.5–25.0 [kg /m^2^], being healthy and at least one year after the last pregnancy. The exclusion criteria included medical contraindications to participate in the study as well as a history of serious orthopaedic or neurological injuries. Furthermore, the subjects did not feature clinically relevant foot deformities, pedal edema, foot pain or neuropathy. All subjects who met the abovementioned inclusion criteria gave signed and informed consent before the beginning of the study. The present research is a part of our longitudinal study of gait in women during and after pregnancy in which 36 healthy pregnant women were initially enrolled [20]. The study was approved by the Regional Bioethics Committee in Krakow (registration no. 139/KBL/OIL/2011). The research was conducted according to the scientific studies ethic principles stated in the Helsinki Declaration.

The experimental sessions were performed in each of the pregnancy periods: P1—in the first trimester (12^th^ gestation week), P2—in the second trimester (25^th^ gestation week) and P3—in the third trimester of pregnancy (36^th^ gestation week). The initial sample comprised 36 women who took part in the first examination (P1). However, 6 of them resigned from continuing participation in the project due to medical contraindications. Thus, 30 women took part in the second (P2) and third (P3) examination: primigravid (19), second pregnancy (8), third pregnancy (3). All pregnancies were singletons. The mean age in the group at the time when the study started was 30.3±3.4 years.

### Study protocol

During each of the three examinations (P1-P3) the same study protocol was used. First, the anthropometric measures were taken. Then walking trials at a self-selected speed along a ~6-m walkway were registered with the FreeMED force platform (Sensor Medica, Italy). All the experimental sessions took place in the morning to avoid influence of tiredness on the studied parameters. The participants were wearing a tight-fitting t-shirt and shorts.

#### Anthropometric measurements

The following anthropometric measurements were taken: BH—body height (Basis–vertex, measured without shoes, in standing position to the nearest 0.1 cm, with the head in the Frankfurt plane, using a stadiometer), BM—body mass (measured to the nearest 0.1 kg, using a clinical balance scale), WC- waist circumference (measured to the nearest 0.1 cm by using an anthropometric tape in the narrowest place on the waist between the lower edge of costal arch and the upper edge of iliac crest with the subjects in standing position, recorded at the end of a gentle expiration). The data quality was assured by an extensive training and all the measurements were taken by the same person (A.S.). BMI was calculated as body weight in kilograms divided by height in meters squared.

#### Assessment of the foot loading pattern

**Fig 1:**
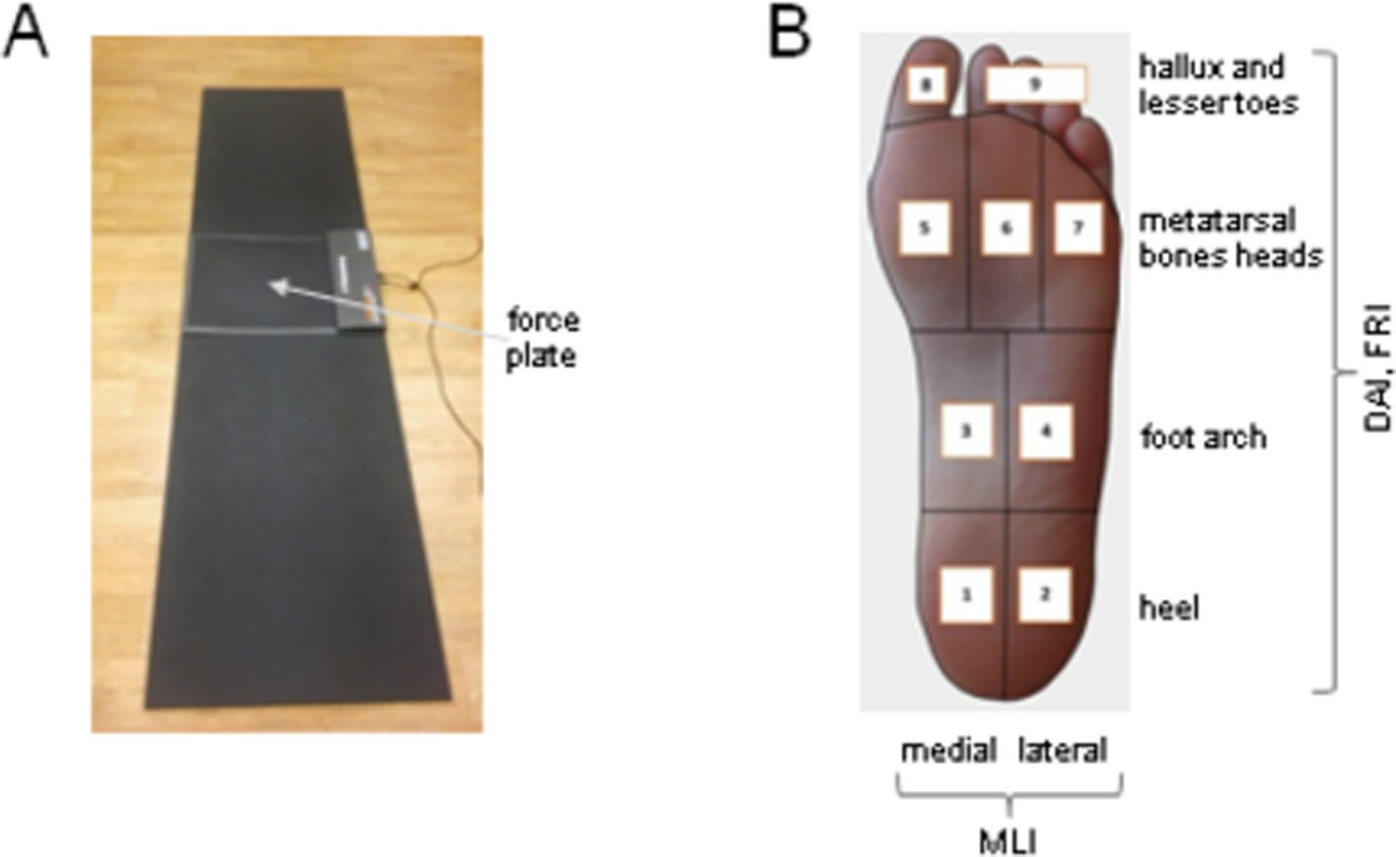
Experimental setup and foot pressure distribution measurements. **A**–the participants were asked to walk barefoot along a ~6-m walkway at comfortable self-selected speeds. Vertical foot pressure was recorded by the force plate (40x40 cm) located in the middle of the walkway. **B**–foot division into 9 regions (to evaluate the corresponding indicators of foot loading—DAI, FRI, MLI): medial (1) and lateral (2) heel; medial (3) and lateral (4) foot arch; first (5), second-third (6) and fourth-fifth (7) metatarsal bones heads; hallux (8) and lesser toes (9).

The subjects were asked to walk along a pathway at self-selected speed while an assessment of the feet loading pattern was performed using the FreeMED force platform (40x40 cm, resistive conductive sensors organized in matrix with 2.5 dpi spatial resolution) located in the middle of the pathway (Fig 1A). The data were sampled at 400 Hz. The self-selected walking speed of the women did not change significantly throughout pregnancy (~1.3 m/s) [20]. The starting point was determined in such a way that, regardless of the step length, the foot could achieve full contact with the platform at least at the third step (‘midgait technique’) [24]. Subjects were instructed to perform several minutes’ walking to warm up, get familiar with laboratory environment and adjust step length. The examination continued until 3 correct footprints for each side were achieved and the averaged results from all 3 obtained footprints were used for the analysis. In order to assess the contact area of the specific foot parts, the picture obtained was automatically divided into 9 regions (medial (1) and lateral (2) heel, medial (3) and lateral (4) foot arch, first (5), second-third (6), and fourth-fifth (7) metatarsal bones heads, hallux (8), and lesser toes (9) (Fig 1B).

The following three indicators were used.

Indicator 1: DAI–dynamic longitudinal arch index was calculated using the approach of Cavanagh and Rogers (1987) as a ratio of medial and lateral foot arch contact area (region 3 and 4) (cm^2^) to a sum of the whole foot contact area, excluding toes (regions 1 to 7) (cm^2^) [25]:

$$\mathrm{DAI}=\frac{3+4({\mathrm{cm}}^{2})}{1+2+3+4+5+6+7({\mathrm{cm}}^{2})}$$

The larger the DAI value, the more flattened the foot arch is.

The foot was also automatically divided into forefoot and rearfoot areas and into medial and lateral parts and their relative loading (in %) during stance was computed (the whole foot constituted 100%). Accordingly, two other indicators of the relative loading of particular areas of the feet were calculated:

Indicator 2: FRI–forefoot and rearfoot loading ratio:

$$\mathrm{FRI}=\frac{\mathrm{Forefoot}(\%)}{\mathrm{Rearfoot}(\%)}$$

Indicator 3: MLI - ratio of medial and lateral foot loading:

$$\mathrm{MLI}=\frac{\mathrm{Medial}\;\mathrm{foot}\;\mathrm{loading}(\%)}{\mathrm{Lateral}\;\mathrm{foot}\;\mathrm{loading}(\%)}$$

These indicators (FRI and MLI) evaluate the relative rather than absolute load of forefoot vs. rearfoot, and medial vs. lateral part of the foot. Finally, the foot angle (FA), the angle between the line running across the foot axis and the line running along the platform axis, was also assessed. All indicators were computed for both right and left foot.

### Statistical analysis

The data obtained were analysed using Statistica 13 (StatSoft) and SPSS statistical software. Descriptive statistics of foot loading indicators included the calculation of the mean and SD. T-test for dependant samples was used to assess foot loading changes throughout pregnancy (differences between the 1^st^, 2^nd^, and 3^rd^ trimesters of pregnancy). The Pearson correlation coefficient was used to analyse relationship between selected anthropometric and foot loading pattern indexes. The correlation coefficient was calculated for the increments of anthropomorphic indexes and the foot loading pattern indicators in relation to the previous trimester. The results were considered significant for *P*<0.05.

## Results

The anthropometric characteristics of the subjects at the three data collection sessions (P1-P3) are presented in Table 1.

**Table 1:** Anthropometric characteristics of women in the 1^st^ (P1), 2^nd^ (P2) and 3^rd^ (P3) trimesters of gestation [mean (SD)].

	P1	P2	P3
**WC**, cm	78.1 (5.3)	97.0 (8.2)	104.2 (6.5)
**BM**, kg	61.5 (6.8)	67.4 (7.4)	72.6 (8.2)
**BMI**, kg/m^2^	21.9 (2.0)	24.0 (2.2)	25.9 (2.7)

WC–waist circumference, BM–body mass, BMI–body mass index.

### Dynamic arch indexes for the right (DAI_R_) and left (DAI_L_) feet in subsequent periods of pregnancy (P1-P3)

**Fig 2:**
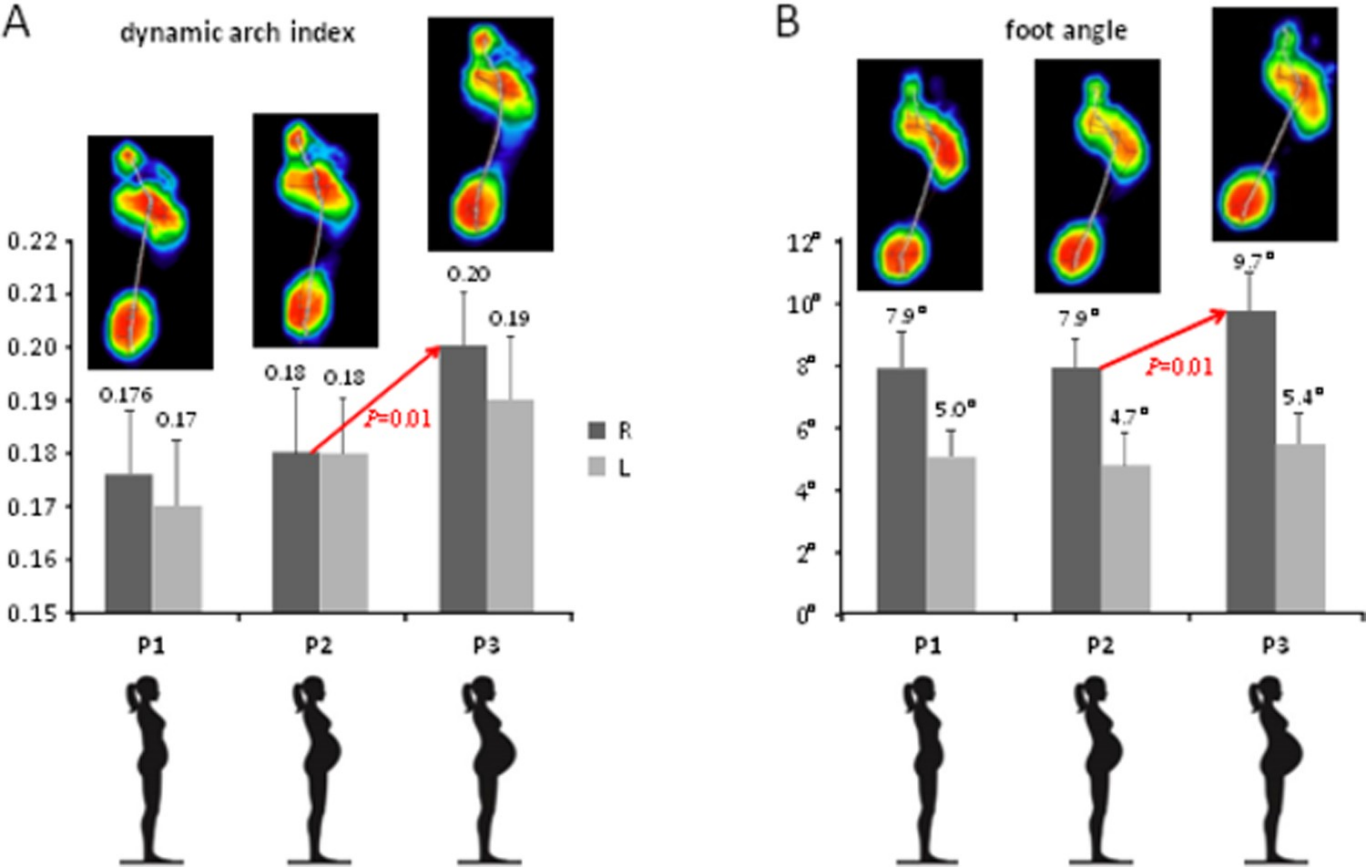
Foot loading characteristics in the 1^st^ (P1), 2^nd^ (P2) and 3^rd^ (P3) trimesters of gestation. **A**–dynamic arch index (mean±SD). **B**–foot angle (mean±SD). Upper colour footprints in A and B illustrate examples of averaged right foot pressure distribution patterns of individual subjects in the 1^st^, 2^nd^ and 3^rd^ trimesters of gestation.

The dynamic arch index characterises foot flattening (the larger the DAI value, the more flattened the foot arch is) and it was on average ~0.17–0.2 in the participants (Fig 2A). An increase in DAI_R_ and DAI_L_ mean values was observed along with the development of pregnancy. The differences proved to be statistically significant only for P3 vs. P2 measurements for the right foot (P = 0.01) (Fig 2A).

### Forefoot-rearfoot (FRI) and medial-lateral (MLI) indexes

The forefoot-rearfoot and medial-lateral indexes for the right and left feet (on average, FRI was ~1.6–1.7, and MLI was ~1) showed a tendency to slightly decrease in P2 compared to P1, followed by an increase in P3, however, these changes did not reach significant differences between pregnancy periods (P>0.05) (S1 and S2 Tables).

### Foot angle for the right (FA_R_) and left (FA_L_) feet in subsequent periods of pregnancy

The foot angle (~5–9°, Fig 2B) tended to increase along with the pregnancy progress. This effect was visible in P3, with the statistical significance in FA_R_ values between P3 and P2 (P = 0.01) (Fig 2B).

### Correlations between changes in the selected anthropometric variables and the distribution of foot loads and placements during pregnancy

The individual anthropometric characteristics vary among participants and their relationship with foot loading patterns during pregnancy was assessed. Although the correlation of individual foot loading parameters across different trimesters was relatively high, more noticeably for DAI and FA and less for the MLI, and FRI measurements (Table 2), significant correlations were found between the anthropometric characteristics and dynamic arch and medial-lateral loading indexes. In particular, arch flattening correlated with the body mass in all trimesters while the medial-lateral loading index correlated only in the first and second trimesters (Table 3). The forefoot-rearfoot loading index was not influenced by the body mass. Waist circumference changes significantly influenced dynamic arch flattening but only in the late pregnancy.

**Table 2:** Correlation coefficients between foot loading parameters (DAI, FRI, MLI and FA) in the 2^nd^ vs. 1^st^ (P2 vs P1) trimester and in the 3^rd^ vs. 1^st^ (P3 vs P1) trimester of pregnancy.

		DAI	FRI	MLI	FA
**P2 vs P1**	R	0.91	0.58	0.58	0.94
	L	0.92	0.76	0.45	0.92
**P3 vs P1**	R	0.90	0.54	0.66	0.91
	L	0.86	0.79	0.50	0.84

DAI–dynamic arch index, FRI–forefoot-rearfoot index, MLI—medial-lateral index, FA–foot angle, R–right, L—left.

**Table 3:** Correlation coefficients between anthropometric data (WC, BM and BMI) and foot loading parameters (DAI, FRI, MLI and FA) in the 1^st^ (P1), 2^nd^ (P2) and 3^rd^ (P3) trimesters of pregnancy.

			DAI			FRI			MLI			FA	
		P1	P2	P3	P1	P2	P3	P1	P2	P3	P1	P2	P3
**WC**	r	0.06	0.46*	0.51*	0.04	0.13	0.11	-0.14	-0.22	-0.12	0.30	0.16	0.10
	*P*	0.73	0.004	0.001	0.81	0.44	0.52	0.39	0.18	0.46	0.07	0.33	0.10
**BM**	r	0.52*	0.46*	0.44*	-0.01	-0.22	-0.13	-0.45*	-0.36*	-0.12	0.05	0.07	-0.05
	*P*	0.001	0.004	0.006	0.94	0.19	0.45	0.005	0.03	0.21	0.79	0.69	0.76
**BMI**	r	0.55*	0.44*	0.50*	0.15	-0.06	0.05	-0.49*	-0.37*	-0.32	-0.03	0.04	-0.03
	*P*	0.000	0.005	0.001	0.36	0.71	0.76	0.002	0.02	0.06	0.85	0.84	0.86

WC–waist circumference, BM–body mass, BMI–body mass index, DAI–dynamic arch index, FRI–forefoot-rearfoot index, MLI—medial-lateral index, FA–foot angle. The data for the left and right foot were pooled together. Asterisks denote significant correlations (P-values are indicated).

## Discussion

We aimed at analysing the successive modifications of the plantar pressure distribution pattern in connection with pregnant women body adjustments, namely body mass and its distribution. To our knowledge, the study is the first longitudinal one in which the relation between abdominal size and plantar pressure distribution pattern was analysed. The correlation of individual foot loading parameters across different trimesters was relatively high, more noticeably for DAI and FA (r~0.9) and less for the MLI, and FRI measurements (r~0.5–0.8) (Table 2). Nevertheless, there were also changes depending on the phase of pregnancy. Plantar pressure alterations which occur during pregnancy may be related to both biomechanical factors and gait adaptations. Below, the longitudinal changes in the main foot loading characteristics are discussed, namely, foot arch flattening, relative foot areas loading, and foot placements.

Foot arch flattening during gait was evaluated by measuring the DAI parameter, which correlated with the body mass in all trimesters (Table 2), consistent with the influence of individual biomechanical factors (e.g. internal loads related to the anatomical structure of the body) on foot loading. An increased body mass generally evokes changes in the height of the foot arch during posture [16, 26, 27]; the higher the weight, the more significant changes can be observed [28], resulting in the increase in midfoot contact area and midfoot plantar pressure in late pregnancy [9, 11, 13]. Body mass in pregnant women increases significantly during relatively short period of time, by an average of 11 to 16 kg [9, 29]. In the presented sample of participants, the mass gain was about 11 kg (Table 1). As far as it concerns the longitudinal changes during gait, the results showed a tendency of longitudinal foot arch flattening for both feet (increasing values of DAI, Fig 2A), however, the observed changes were statistically significant only for the right foot when comparing P2 and P3.

Not only mass gain but also body mass distribution can be linked to plantar pressure changes. About half of body mass gained during pregnancy is situated in the abdominal area (anterior part of the trunk) which leads to changes in the centre of gravity and greater oscillations of the centre of pressure [2, 5, 15]. The compensations, which are believed to follow the centre of gravity deviation, include increased lumbar lordosis, sagittal pelvic tilt and a more posterior upper body tilt [30, 31]. Also, forward shift of plantar loading has been reported [12]. Bertuit et al. (2016) showed no difference in the plantar pressure distribution between women in the last 4 months of pregnancy and a control group, which may indicate that the adaptations had taken place before. Ribeiro et al. [9] in their longitudinal study described gradually increasing loads of the forefoot (maximum force and peak pressure) and reduction in the rearfoot. Otherwise, an increased rearfoot and a decreased forefoot peak pressure in the course of pregnancy was observed [18].

One of the objectives of our study was to examine how the anthropometric characteristics may influence the foot loading pattern depending on the phase of pregnancy. In particular, we found that while foot arch flattening correlated with the body mass in all trimesters (as mentioned above), the medial-lateral loading index correlated only in the first and second trimesters (Table 3). The forefoot-rearfoot loading index was not influenced by the body mass. Waist circumference changes significantly influenced dynamic arch flattening but only in the late pregnancy (P2 and P3, Table 3). In the third trimester of pregnancy, a small though significant increase in the right foot angle was also observed (Fig 2B). Karadag-Saygi et al. [12] showed greater loading of the right forefoot in pregnant women during walking, however, their sample comprised women in the 3^rd^ trimester of pregnancy. Nevertheless, we also revealed a slight ‘asymmetrical’ adaptation of foot placement characteristics in the sample of right-leg dominant women (greater DAI in P3 for the right foot, Fig 2A, and greater right foot angles, Fig 2B). While these changes were relatively small, they might be functional constituting body adaptation to remain stable besides pregnancy related anthropometric changes. Functional asymmetry has been defined as a consistent task discrepancy between the two lower limbs. Within the concept of the limb dominance, the non-dominant lower limb contributes more to support, while the dominant lower limb contributes more to forward propulsion [23]. For instance, other examples are known of subtle but functional asymmetries during stepping [32], or when gait asymmetries, not evident during normal walking, appear during more challenging walking tasks [33].

Mass gain and the ventrally driven centre of gravity induce gait disturbances in a pregnant woman [17, 34]. As the literature revealed adaptations following pregnancy are recognised to provide safety and stability [e.g. 9, 35, 36]. The most important features identified by the authors are as follows: reduced walking velocity as a result of lower frequency and smaller length of the steps, longer stance time and increased stance width compensated by medio-lateral component of GRF. Additionally, considering the lower limbs adjustments the most affected by the continuous overloads in the course of pregnancy occurred to be a hip joint [37, 38] as being closer to the body region with greater anatomical and morphological changes [37]. As mentioned, to improve gait stability pregnant women walk with a wider support base [17, 20], which is especially visible in the third trimester of pregnancy [1, 39]. The base of support can be wider both due to increased distance between the ankles, and as a result of a greater foot angle. While Foti et al. [38] showed that an external foot progression angle remained unchanged during pregnancy, in our longitudinal study it was found that the angle of the foot tends to increase with the advancement of pregnancy, though changes in the foot angle were significant only for the right foot (Fig 2B).

Our findings showed that individual anthropometric characteristics affect plantar pressure distribution in pregnant women (Table 3). However, they also revealed modifications or adaptations that depend on the period of pregnancy, e.g., significant correlations of MLI in the 1^st^ and 2^nd^ trimesters but not in late pregnancy. Plantar pressure distribution changes may play a role in improving gait stability in the stance phase [9]. For instance, Mei et al. [13] suggested that flattening of the medial longitudinal arch can result in a decreased stability during pregnancy, therefore, the observed increase in the foot angle on the same side as medial arch collapse may constitute the adaptation to keep gait stability despite medial arch collapse. This might be especially important since P3 is the time of pregnancy when the incidence of falls resulting in hospitalization is the largest and concerns almost 80% of pregnant women [6]. Although none of the examined women claimed to suffer from falls, it can add value to future research concerning the factors influencing greater risk of falling in the last trimester of pregnancy and its relation to individual anthropometric characteristics or gait adaptations. Another interesting factor, which can possibly influence foot loading distribution changes in pregnancy, is parity. In the study the majority of women were primigravid and thus this factor was not taken into consideration. However, it will be interesting in the future to investigate whether plantar pressure distribution changes that occur in the course of pregnancy differ in first and subsequent pregnancies. Furthermore, it is of great importance to see pregnancy as the time of continuous changes that may begin right from the beginning, i.e. from the 1^st^ trimester. In our previous publication, concerning a group of 15 women, it was demonstrated that feet loading pattern during gait was not altered throughout the first trimester of gestation compared to time before pregnancy, however, the size of the base of support (reflecting feet placement) significantly increased [40]. Bearing this in mind, it is advisable to arrange the related studies starting from before pregnancy to have reference point for the subsequent pregnancy periods.

## Conclusions

The findings provided the characteristics of the relative foot areas loading throughout pregnancy in relation to anthropometric features of the woman’s body. With the advancement of pregnancy the risk of medial arch flattening increases driven by body mass gain, which can result in less stable gait. Our results showed that pregnant women cope with decreased stability during gait by repositioning of their feet (manifested in increased foot angle) to enhance gait stability.

## Supporting information

S1 Table Forefoot-rearfoot index (FRI) in subsequent trimesters of pregnancy (P1-P3) for right and left feet. FRI–forefoot-rearfoot index, P1-P3 – 1^st^ (P1), 2^nd^ (P2) and 3^rd^ (P3) trimesters of pregnancy.
(DOC)

S2 Table Medial-lateral index (MLI) in subsequent trimesters of pregnancy (P1-P3) for right and left feet. MLI–medial-lateral index, P1-P3 – 1^st^ (P1), 2^nd^ (P2) and 3^rd^ (P3) trimesters of pregnancy.
(DOCX)
