# Supplementary material for: Influence of pregnancy related anthropometric changes on plantar pressure distribution during gait—A follow-up study
Source: PLoS One. 2022 Mar 11;17(3):e0264939. doi: 10.1371/journal.pone.0264939 (PMC8916641; doi:10.1371/journal.pone.0264939)
Supplement: S1 Table — FRI–forefoot-rearfoot index, P1-P3 – 1st (P1), 2nd (P2) and 3rd (P3) trimesters of pregnancy. (DOC) [file pone.0264939.s001.doc]

**S1 Table.** **Forefoot-rearfoot index (FRI) in the 1^st^ (P1), 2^nd^ (P2) and 3^rd^ (P3) trimesters of pregnancy for right and left feet.**

| **Compared pregnancy periods** | **(FRI)Difference of average values** | **T** | **p** |
| --- | --- | --- | --- |
| **P1 vs P2 - right**  **P1 vs P2 - left** | -0.01 | -0.11 | 0.91 |
|  | -0.01 | -0.37 | 0.71 |
| **P2 vs P3 - right**  **P2 vs P3 - left** | -0.03 | -1.01 | 0.32 |
|  | -0.07 | -1.75 | 0.10 |

FRI – forefoot-rearfoot index, P1-P3 – 1^st^ (P1), 2^nd^ (P2) and 3^rd^ (P3) trimesters of pregnancy.
