# Supplementary material for: Influence of pregnancy related anthropometric changes on plantar pressure distribution during gait—A follow-up study
Source: PLoS One. 2022 Mar 11;17(3):e0264939. doi: 10.1371/journal.pone.0264939 (PMC8916641; doi:10.1371/journal.pone.0264939)
Supplement: S2 Table — MLI–medial-lateral index, P1-P3 – 1st (P1), 2nd (P2) and 3rd (P3) trimesters of pregnancy. (DOCX) [file pone.0264939.s002.docx]

**S2 Table. Medial-lateral index (MLI) in the 1^st^ (P1), 2^nd^ (P2) and 3^rd^ (P3) trimesters of pregnancy for right and left feet.**

| **Compared pregnancy periods** | **(MLI)Difference of average values** | **T** | **p** |
| --- | --- | --- | --- |
| **P1 vs P2 - right**  **P1 vs P2 - left** | 0.07 | 1.10 | 0.06 |
|  | -0.00 | -0.03 | 0.97 |
| **P2 vs P3 - right**  **P2 vs P3 - left** | -0.00 | -0.08 | 0.93 |
|  | -0.04 | -0.88 | 0.39 |

MLI – medial-lateral index, P1-P3 – 1^st^ (P1), 2^nd^ (P2) and 3^rd^ (P3) trimesters of pregnancy.
